# Quantifying guideline-discordant intermittent catheterization in adults hospitalized with spinal cord injury: a retrospective cohort study

**DOI:** 10.1038/s41393-025-01078-w

**Published:** 2025-04-29

**Authors:** Mengdong He, Emily Hon, Lin Xu, Stephen Hampton, Kimberly Waddell

**Affiliations:** 1 David Geffen School of Medicine at the University of California, Los Angeles, Los Angeles, CA USA; 2 Department of Physical Medicine and Rehabilitation, University of Pennsylvania, Philadelphia, PA USA; 3 Division of General Internal Medicine, Perelman School of Medicine, University of Pennsylvania, Philadelphia, PA USA; 4 Penn Medicine Nudge Unit, University of Pennsylvania, Philadelphia, PA USA; 5 Corporal Michael J. Crescenz VA Medical Center, Philadelphia, PA USA

**Keywords:** Health services, Spinal cord diseases, Spinal cord diseases, Outcomes research

## Abstract

**Study Design:**

Retrospective cohort study.

**Objectives:**

To characterize guideline-discordant clean intermittent catheterization (CIC) during hospitalizations of patients with spinal cord injury (SCI), explore predictors of guideline-discordant CIC, and examine its association with urinary tract infection (UTI).

**Setting:**

Acute care hospitals within a large academic health system.

**Methods:**

Using electronic health records (9/1/2021-9/30/2023), we identified adults hospitalized with a discharge diagnosis of SCI and ≥1 documented CIC bladder output. The primary outcome was guideline-discordant CIC (bladder output volume >500 mL and/or time between CIC > 6 h). Generalized linear model and Chi-square test were used to evaluate patient factors and UTI risk associated with guideline-discordant CIC.

**Results:**

The study included 413 patients with SCI covering 8,016 CIC measurements during 519 hospitalizations. Their mean (SD) age was 55.2 (20.6) years, with 34.7% female and 46.8% Black. 52.8% were covered by Medicare. 79.4% had a thoracolumbar-level SCI. 50.2% of CICs were guideline-discordant. Males and those with managed care insurance had significantly higher odds of guideline-discordant CIC (OR = 1.34, 95% CI, 1.03 to 1.73 and OR = 2.05, 95% CI, 1.18 to 3.54, respectively). Patients with an indwelling catheter for ≥12 days before initiating CIC had significantly lower odds of guideline-discordant CIC (OR = 0.65, 95% CI, 0.49 to 0.84). The UTI incidence was 12.5% in hospitalizations with guideline-discordant CIC compared to 10.4% with guideline-concordant CIC (*P* = 0.49).

**Conclusions:**

Half of CICs did not adhere to guidelines, highlighting the need for quality improvement initiatives. Further research examining the association between UTI and CIC care patterns is warranted.

## Introduction

Spinal cord injury (SCI) often results in significant morbidity and disability, requiring comprehensive management strategies for multifaceted complications [1]. It is estimated that approximately 305,000 persons are living with a SCI in the United States, with an annual incidence of 18,000 new cases [2]. Among the various challenges faced by individuals with SCI, neurogenic bladder is one of the most critical and persistent phenomena [3, 4]. Ongoing bladder management is crucial in the acute and chronic care of this population to prevent medical complications, particularly urinary tract infections (UTIs) or autonomic dysreflexia, and adverse patient outcomes [5, 6].

During an acute hospitalization, bladder management decisions are often complex and influenced by many factors. For appropriate patients, clean intermittent catheterization (CIC) is often preferred over an indwelling urinary catheter (IUC) due to its association with better bladder compliance and fewer urologic complications such as UTIs [7, 8]. Clinical guidelines for CIC emphasize the importance of regular catheterization intervals, typically every 4–6 h, and maintaining bladder volumes below 500 mL to minimize the risk of bladder overdistention and associated complications such as autonomic dysreflexia or acute kidney injury [5, 6, 9, 10]. Because catheter associated UTIs are one of the most common healthcare-associated infections, many health systems prioritize the early removal of IUCs as a key prevention strategy [11]. This has likely resulted in the initiation of CIC, for appropriate patients, much earlier in the recovery process. This shift highlights the importance of adhering to CIC guidelines to optimize patient outcomes and prevent medical complications associated with poor bladder management among individuals with SCI.

Despite the established benefits and best practice of CIC, there is a significant gap in the literature regarding the characterization of CIC practices during an acute hospitalization for patients with SCI. Previous research has predominantly focused on comparing different bladder management strategies during the acute hospitalization for SCI and their associations with urological complications [12–14] or exploring long-term compliance, patient satisfaction, or changes in bladder management strategies after discharge, linking these factors to health outcomes [15–19]. There is a lack of comprehensive data on whether CIC practices during hospitalizations adhere to established guidelines and how such adherence impacts outcomes. This gap underscores the need for a detailed examination of CIC practices in the acute care setting to inform strategies enhancing adherence to clinical guidelines, thereby improving the quality of care for patients with SCI.

The primary objective of this study was to quantify the prevalence of guideline-discordant CIC management among patients hospitalized with SCI, which will establish baseline care patterns. Secondly, this study explored predictors of guideline-discordant CIC practices. Understanding the patient and clinical factors associated with guideline-discordant CIC is essential for developing targeted interventions or quality improvement initiatives that promote adherence to clinical guidelines. Lastly, the study examined the association between guideline-discordant CIC and the incidence of UTIs during hospitalization. Elucidation of this relationship may help healthcare providers implement more effective bladder management protocols, ultimately enhancing patient outcomes and reducing the burden of urinary complications among adults with SCI. By addressing these objectives, this study aims to fill the existing gaps in the literature and provide valuable insights into optimizing CIC bladder management for individuals with SCI in the acute care setting.

## Methods

This was a retrospective cohort study examining guideline-discordant CIC among patients with SCI hospitalized within a large, academic healthcare system. The study included all admissions between September 1, 2021 to September 30, 2023, across five acute care hospitals located in diverse demographic regions (three urban and two mid-size area). Patients were eligible for study inclusion if the following criteria were met: (1) ≥ 18 years of age; (2) a discharge diagnosis of SCI; and (3) at least one documented CIC bladder output volume during the hospital admission. A SCI discharge diagnosis was identified using International Classification of Diseases 10th revision (ICD-10) diagnosis codes (Supplement Table S1). Patients were excluded if they had a hospital stay <24 h or died during hospitalization. The University of Pennsylvania Institutional Review Board approved this study with a waiver for informed consent due to the retrospective design.

### Data sources and cohort

Data was extracted from electronic health records (EHR) using the Epic Clarity database. Epic Clarity stores both patient demographic and clinical data. Demographic information included age, sex, race, ethnicity, and insurance type at admission. Clinical data elements, such as discharge diagnoses, were extracted for each eligible admission. To obtain CIC-associated bladder output volumes, both medical orders and flowsheet data were extracted. Specifically, patient bladder output type (e.g., CIC or IUC), volume, and timestamp were identified by using output labels in the flowsheet data (Supplemental Table S2). For example, if a bladder output was labeled as “intermittent/straight cath (mL)”, it was classified as a CIC occurrence.

For bladder output measurements with non-specific labels, such as “urine output”, we referenced the clinician medical order file to determine if a bladder output was from CIC **(**Supplemental Table S2). For example, if there was an active medical order for straight catheterization at the time of the recorded non-specific bladder output, the bladder output volume was re-classified as CIC-associated. Similarly, if there was an active medical order for insertion or maintenance of IUC, the non-specific bladder output volume was re-classified as IUC-associated. The IUC-associated bladder output records were retained in order to calculate the duration of IUC use prior to the initiation of CIC, which was examined as a predictor for guideline-discordant CIC. In the case where both CIC and IUC orders were active, we used the order placed closest in time to the bladder output measurement. If none of the CIC and IUC related orders were active, the bladder output volume was kept as a non-specific urine output, not associated with either CIC or IUC, and excluded from this analysis.

### Outcome assessment

The primary outcome was guideline-discordant CIC management. Guideline discordance was defined as a CIC-associated bladder output volume >500 mL and/or a time interval between CIC occurrences >6 h [5, 6]. Using these criteria, each CIC occurrence was labeled as either guideline-discordant or guideline-concordant.

We calculated the time interval between consecutive CIC occurrences by using the timestamps of CIC-associated bladder output volumes that were recorded during the same admission. The time intervals between CICs were capped at 24 h, as CIC is typically completed multiple times a day. A set of CIC measurements where each measurement was less than 24 h from the prior one was considered a continuous bladder management sequence and defined as a “CIC stretch”. By capping at 24 h, we calculated time intervals between consecutive CICs within the same CIC stretch, but not ones from different CIC stretches, to account for medical teams trialing different bladder management methods during the admission. In the scenario where there was only one CIC recorded during the admission, CIC intervals could not be calculated, and thus guideline discordance was assessed solely based on the bladder output volume.

The secondary outcome was UTI. A UTI case was identified using a combination of ICD-10 diagnosis codes (Supplement Table S3), abnormal urine culture, and administration of antibiotics (cefpodoxime, trimethoprim/sulfamethoxazole, levofloxacin, or cefepime) [20, 21]. The administration time of antibiotics was required to be during the urine culture collection time window plus 24 h. If a patient met the definition of UTI at any point during the admission, they were classified as a UTI case. When multiple UTI events were identified during the same admission, we used the first event in the analysis, meaning each patient admission was allowed to have up to one UTI event.

### Statistical analyses

Patient characteristics at admission and descriptive analyses of guideline-discordant CIC management were summarized by mean (standard deviation, SD) or median (interquartile range, IQR) for continuous variables and by count and percentages for categorical variables.

To examine factors associated with guideline-discordant CIC management, we fit a generalized linear model with nested random effects for admission and patient to adjust for correlation of CIC recordings within the same admission and patient. The patient factors included age, sex, race, and insurance type at admission. Clinical factors included level of spinal cord injury (cervical versus thoracolumbar level), duration (in days) of IUC prior to CIC, time of day when CIC was completed (daytime = 7am-7pm, or nighttime = 7pm-7am). Additionally, the model adjusted for intravenous fluid infusion that overlapped with or stopped within 24 h before CIC occurrence, as this may increase bladder output volume and lead to a higher chance of guideline-discordant CIC. Beta coefficients from the model were exponentiated to obtain odds ratios (OR) and 95% confidence intervals (CI).

Differences in UTI incidence by guideline-discordant status were examined using a Chi-square test. Because the incidence of UTI was assessed only once during an admission and CICs could occur multiple times during the same hospitalization, we created a variable for guideline discordance at the admission level. An admission was classified as having guideline-discordant CIC if the median bladder output volume, across all recorded outputs, was >500 mL and/or the median time between CIC sessions, across all CIC sessions, was >6 h during the admission. All analyses were implemented using SAS statistical software version 9.4.

## Results

**Fig. 1:**
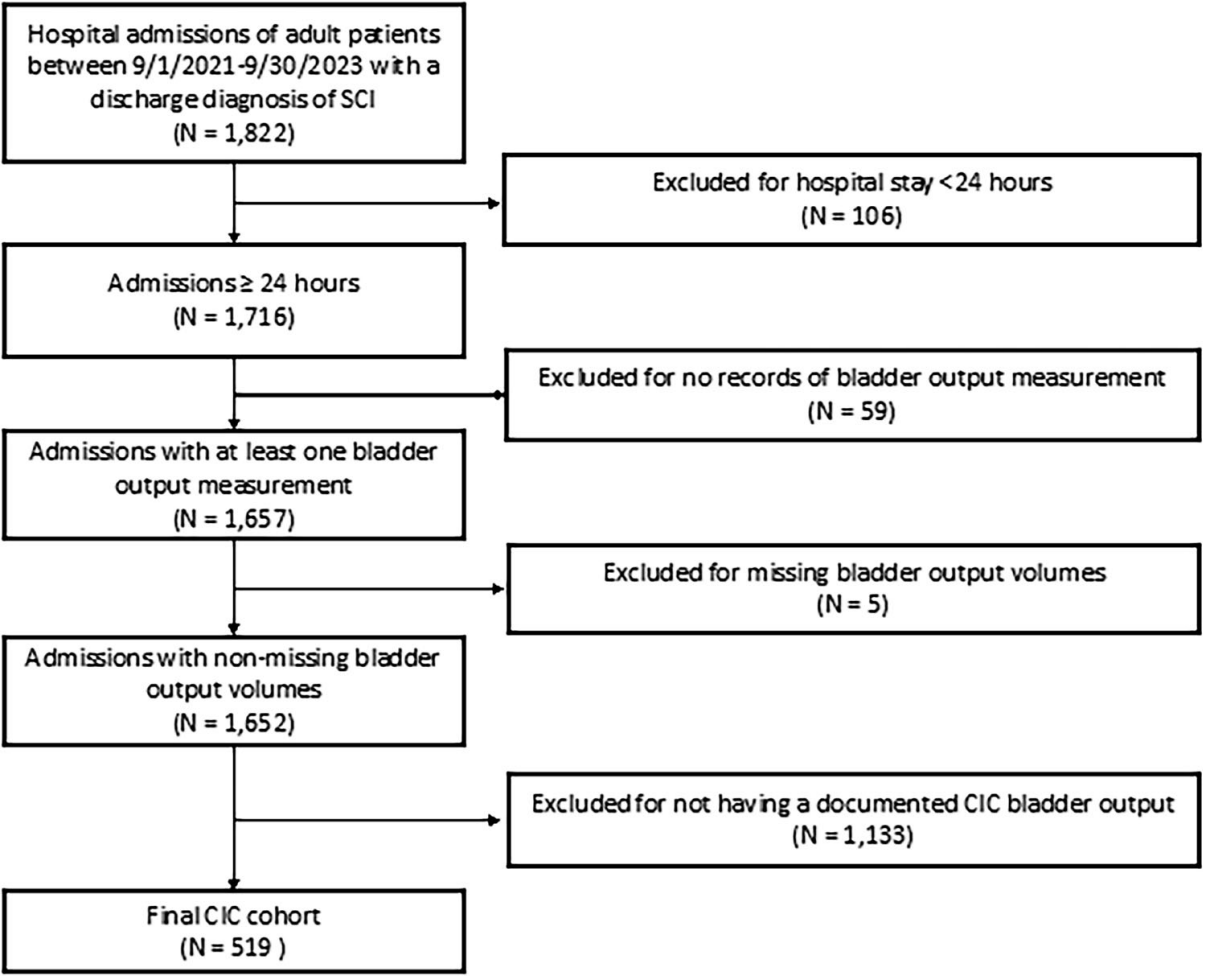
Cohort formation. CIC clean intermittent catheterization, *N* number of admissions, SCI spinal cord injury.

The final sample included 519 admissions and 413 unique patients (Fig. 1). There were 8,016 CIC measurements included in the analysis. The mean (SD) age of the study sample was 55.2 (20.6) years and 34.7% were female. Overall, 46.8% of the sample self-reported as Black race and 43.9% were White race. Approximately half of the patient admissions were covered by Medicare (52.8%) and a quarter by Medicaid (28.9%). Most SCI cases were due to injuries below the cervical level (79.4%). Overall, half of the included hospitalizations in the study sample received both CIC and IUC (50.1%) for bladder management while the other half received only CIC (49.9%) (Table 1). 16.8% of CICs (1,345 measurements) took place during intravenous fluid infusion or within 24 h after infusion ended.

**Table 1:** Sample demographics.

	Overall cohort (*N* = 519)
Age, years, mean (SD)	55.2 (20.6)
Sex	
Female	180 (34.7%)
Male	339 (65.3%)
Race	
White	228 (43.9%)
Black	243 (46.8%)
Asian	9 (1.7%)
Other	39 (7.5%)
Ethnicity	
Hispanic Latino	17 (3.3%)
Not Hispanic or Latino	498 (96.0%)
Unknown	4 (0.7%)
Insurance type	
Medicare	274 (52.8%)
Medicaid	150 (28.9%)
Private	44 (8.5%)
Managed care	51 (9.8%)
SCI type	
Cervical level	107 (20.6%)
Thoracolumbar level	412 (79.4%)
Bladder management strategy	
CIC only	259 (49.9%)
CIC and IUC	260 (50.1%)

*CIC* clean intermittent catheterization, *IUC* indwelling urinary catheter, *N* number of admissions, *SCI* spinal cord injury, *SD* standard deviation.

**Fig. 2:**
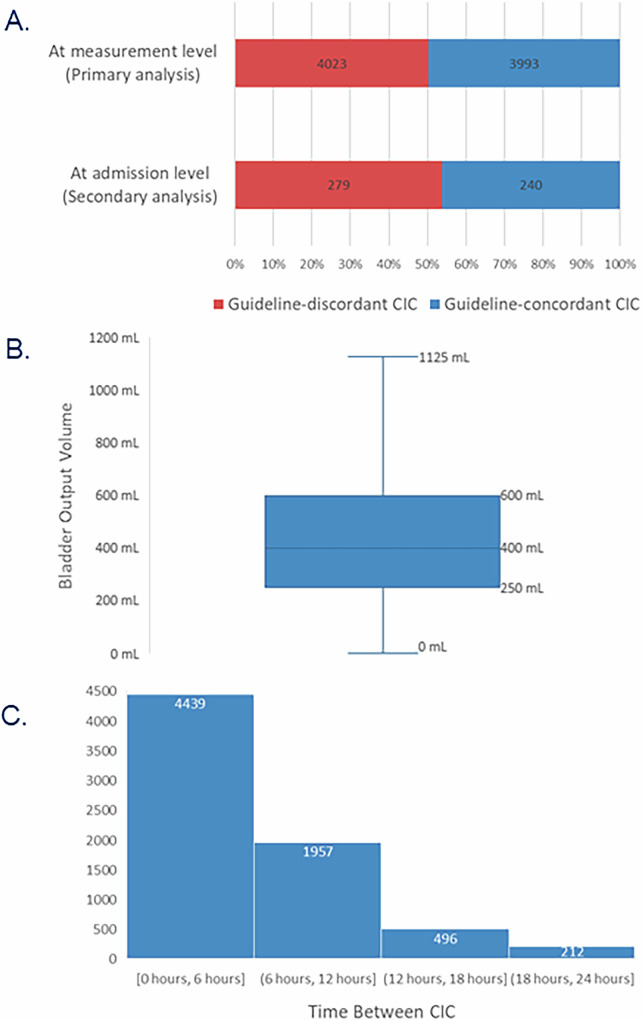
Key features of CIC management. CIC management included: **A**. Distribution of guideline-discordant CIC; **B**. Distribution of bladder volumes across all CIC occurrences; **C**. Distribution of time between CIC occurrences. Outlier data were excluded from Fig. 2B. CIC clean intermittent catheterization.

Across all 8,016 CIC measurements, 50.2% (4,023 measurements) were identified as guideline-discordant (Fig. 2A). At measurement level, the median [IQR] CIC-associated bladder output volume was 400 [350] mL (Fig. 2B) and the median [IQR] time between CIC measurements was 6 [4] hours (Fig. 2C).

**Fig. 3:**
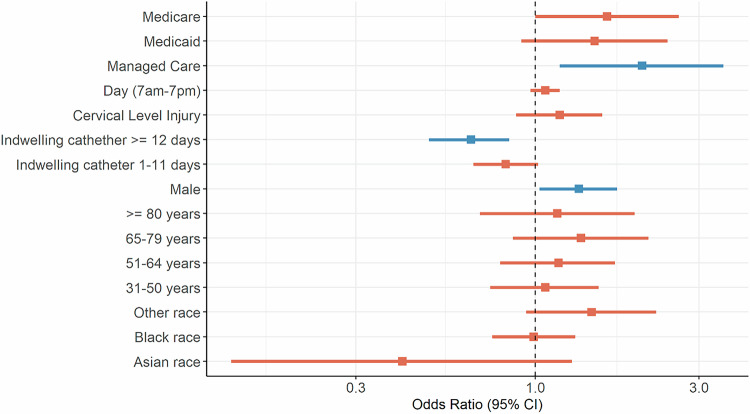
Predictors for guideline-discordant CIC. Reference groups: Private insurance (for Medicare, Medicaid, and Managed Care); Nighttime = 7pm-7am (for daytime = 7am-7pm); Thoracolumbar level injury (for cervical level injury); 0 indwelling catheter days (for indwelling catheter ≥12 days and indwelling catheter ≥1 days); Female (for male); 18–30 years (for 31–50 years, 51–64 years, 65–79 years, and ≥80 years); White race (for Asian, Black, and Other race). Abbreviations: CIC clean intermittent catheterization.

Results from the generalized linear model demonstrated a significant relationship between sex, insurance type, and duration of IUC prior to CIC initiation with guideline-discordant CIC management (Fig. 3, Table S4). Patients who were male, relative to female, had higher odds of receiving guideline-discordant CIC (OR = 1.34, 95% CI, 1.03 to 1.73, *P* = 0.03). Patients covered by managed care insurance, relative to private insurance, also had significantly higher odds of guideline-discordant CIC management (OR = 2.05, 95% CI, 1.18 to 3.54, *P* = 0.01). Lastly, longer duration (≥ 12 days) of IUC use, relative to 0 days, prior to CIC initiation was significantly associated with lower odds of guideline-discordant CIC (OR = 0.65, 95% CI, 0.49 to 0.84, *P* = 0.001).

Although failing to reach statistical significance, it is worth noting that the odds of receiving guideline-discordant CIC were near equal between patients who were Black or White race (OR = 0.99, 95% CI, 0.75 to 1.31). Enrollees of Medicare and Medicaid were more likely to receive guideline-discordant CIC management relative to those with commercial insurance (OR = 1.62, 95% CI, 1.00 to 2.62 for Medicare and OR = 1.49, 95% CI, 0.91 to 2.43 for Medicaid). Being older than 65 years (OR = 1.36, 95% CI, 0.86 to 2.14) and having a cervical level SCI (OR = 1.18, 95% CI, 0.88 to 1.57) were both associated with a slightly higher risk of experiencing guideline-discordant CIC, although not statistically significant. Odds ratios for all predictors are reported in Supplemental Table S4.

Lastly, results from the Chi-Square analysis demonstrated no significant difference in the incidence of UTI between admissions with guideline-discordant CIC and those with guideline-concordant CIC (*P* = 0.4, Table 2). During the 279 hospitalizations that were classified as having guideline-discordant CIC, there were 35 UTI events (12.5%, Fig. 2C). In contrast, during the 240 hospitalizations classified as having guideline-concordant CIC, there were 25 UTI cases (10.4%).

**Table 2:** Risk of UTI by CIC group.

	Number of admissions	Number of UTI	% of UTI	P value^a^
Guideline-concordant CIC	240	25	10.4%	0.45
Guideline-discordant CIC	279	35	12.5%	

*CIC* clean intermittent catheterization, *UTI* urinary tract infection.

^a^Chi-square test.

## Discussion

This study quantified the prevalence of guideline-discordant CIC among patients with SCI hospitalized in a large academic health system and explored the predictors associated with these practices. Our findings revealed that guideline-discordant CIC management is common in the acute hospital setting, with approximately 50% of CIC measurements exceeding the recommended bladder output volume or time interval. Males and/or managed care enrollees had a higher chance of receiving guideline-discordant CIC, while IUC use ≥12 days prior to CIC was associated with a lower chance of experiencing CIC not adherent to guidelines. No statistically significant difference was found in the UTI risk between the guideline-discordant and guideline-concordant CIC groups.

The finding that half of all CIC measurements were guideline-discordant underscores the challenges of adhering to clinical guidelines in acute hospital settings. The medical acuity and complexity of patients in these settings often make it difficult to consistently follow strict guidelines. Multiple medical conditions could be managed simultaneously, and frequent medication changes can impact bladder function and the timing of catheterizations. Transitions between care teams, such as from the ICU to a trauma unit, can lead to unintentional lapses in communication and continuity of care. Additionally, patients may not always be emotionally or mentally ready to engage in the CIC process, placing the responsibility on the care team to remember and perform catheterizations regularly. There is also significant variability across medical teams in how they interpret and apply clinical guidelines for CIC practice. In a survey of nursing practice related to CIC, 46% of the surveyed nurses indicated they based their practice on policies of their facilities, while 25% indicated they relied on their own best practice [22]. Our findings, when put into context, emphasize the value of quality improvement initiatives designed to support healthcare teams in adhering to clinical guidelines more effectively. For example, interventions such as nursing staff education on standardized CIC protocols, automated EHR reminders for timely CIC execution and documentation, and a dedicated EHR datasheet consolidating past and scheduled CIC events may enhance coordination among nursing staff and adherence to guidelines. Once implemented, evaluating the impact of these quality improvement initiatives is essential to understanding what is most effective for facilitating guideline-adherent bladder management and how it affects patient outcomes.

We identified several factors, including sex, insurance type, and the duration of IUC use prior to CIC initiation, that were significantly associated with guideline-discordant CIC management. These findings emphasize the complex interplay between patient and clinical factors in CIC guideline adherence. Individuals with managed care insurance experienced significantly higher odds for guideline discordant CIC, relative to those with commercial insurance. This warrants further investigation to understand what factors may be contributing to guideline-discordant bladder management for those with managed care insurance and how this could be assuaged with changes at the individual, hospital, and policy levels. Interestingly, a longer duration of IUC use before switching to CIC was associated with lower odds of guideline discordance. The association between longer IUC use and lower odds of guideline-discordant CIC may suggest that patients transitioning from prolonged IUC use receive more comprehensive education and support on CIC management. Alternatively, they may have had more time to be stabilized medically or to have other medical conditions that may impact bladder output addressed. Contrary to previous findings that Black patients were less likely to receive IUC and CIC compared to White patients, our study showed a comparable likelihood of experiencing non-adherence to CIC guidelines among Black and White patients in the acute care settings [23].

The incidence of UTI in our study cohort was about 12%, which is lower than the previously reported incidence rate ranging between 40–60% [24, 25]. This reduction may be attributed to the significant efforts healthcare systems have dedicated to UTI prevention, particularly those associated with urinary catheters. Many healthcare systems have implemented policies and procedures, such as early removal of indwelling catheters, aseptic insertion techniques, and regular monitoring of catheter use to mitigate UTI risks. These measures, along with staff education and adherence to infection control protocols, have been pivotal in reducing UTI rates [11, 26, 27]. This difference could also result from the more stringent UTI definition in our study by requiring a combination of UTI diagnosis codes, positive urinary culture, and antibiotic use. Although the incidence of UTIs was slightly higher in the guideline-discordant CIC group compared to the guideline-concordant group (12.54% versus 10.42%), this difference was not statistically significant. This finding may suggest that while guideline-discordant CIC is prevalent, it may not directly translate to a significantly higher risk of UTIs within this study population. However, it’s important to note that this finding was based on an unadjusted analysis. The low number of UTI events in our sample made it statistically challenging to fit a multivariate model that adjusted for relevant covariates (e.g., baseline health status, immunosuppressant use or concurrent urinary tract conditions) that is key for obtaining accurate point estimates. Therefore, this study serves as an initial step in understanding the relationship between guideline-discordant CIC and UTI risk. Future research should focus on conducting adjusted analyses when sample sizes allow, to further investigate the observed trend towards a higher UTI rate in the guideline-discordant group.

To our knowledge, this is one of the first studies to quantify CIC management in a large cohort of patients with SCI during an acute hospitalization. However, several limitations should be acknowledged. First, this study examined patients in a single academic health system. The five acute care hospitals within this healthcare system serve a diverse population in terms of age, race, and insurance types across urban and mid-size areas. However, differences in clinical practices, patient demographics, and resource availability across healthcare systems may limit the generalizability of our results to other healthcare environments. For example, a prior study reported that 14% CIC were conducted at a time interval >6 h and 26% had a bladder output volume >500 mL in sample of 70 patients with SCI in Australia [24]. This underscores the importance of scaling this evaluation to include multiple health systems that range in size, geography, and patient populations. Our findings provide a strong foundation for supporting future, larger evaluations to enhance generalizability. Second, there could be misclassifications in secondary analyses of EHR data. While efforts were made to accurately classify CIC-associated bladder output volumes and timing, there remains a potential for misclassification, especially for non-specific urine output measurements. To mitigate this risk, we used both the flowsheet data as well as clinician medical orders to corroborate bladder outputs, but this approach may not eliminate all threats to validity. Future research could address this limitation by modifying existing EHR documentation to integrate structured data fields for more accurate CIC documentation. Third, residual confounding may be present in our assessment of predictors for guideline-discordant CIC. We used a generalized linear model with nested random effects for admission and patient data to adjust for correlations within the same admission and patient as well as potential key confounders. However, certain systemic factors that could influence CIC practices, such as patient education, nursing CIC protocols, and organizational factors, were not captured in EHR data and thus could not be included in our analysis. Last, only unadjusted analysis was conducted to evaluate the risk of UTI associated with guideline-discordant CIC. Institutional and patient-level factors that contribute to the development of UTI should be included in future research when such data are available and the sample size is sufficient for multivariate adjustment. Additionally, it would be valuable to examine the adjusted relationship between guideline-discordant CIC and other adverse outcomes beyond UTIs, such as autonomic dysreflexia and renal complications through longitudinal studies.

In conclusion, this study highlights the significant prevalence of guideline-discordant CIC management among patients with SCI in acute care settings and identifies key factors associated with non-adherence to clinical guidelines. Addressing these factors through targeted interventions to improve provider care coordination and patient education and support may strengthen adherence to CIC guidelines and ultimately improve clinical outcomes for patients with SCI. Further research is needed to examine the adjusted relationship between guideline-discordant CIC practices and patient outcomes including UTI and other SCI sequelae such as autonomic dysreflexia and to develop comprehensive strategies for optimizing bladder management in this vulnerable population.

## Supplementary information

Supplemental Material

## Data Availability

The data used for the study cannot be shared publicly and may be available from the corresponding author at Penn Medicine upon reasonable request.
