# Supplementary material for: Quantifying guideline-discordant intermittent catheterization in adults hospitalized with spinal cord injury: a retrospective cohort study
Source: Spinal Cord. 2025 Apr 29;63(5):270–6. doi: 10.1038/s41393-025-01078-w (PMC12074987; doi:10.1038/s41393-025-01078-w)
Supplement: Supplementary file 1 — Supplemental Material [file 41393_2025_1078_MOESM1_ESM.pdf]

Table S1. ICD-10 diagnosis codes for SCI definition

ICD-10 diagnosis code	Code description
G82*	Paraplegia (paraparesis) and quadriplegia (quadriparesis)
S12.0	Fracture of first cervical vertebra
S12.2	Fracture of third cervical vertebra
S13.0	Traumatic rupture of cervical intervertebral disc
S13.2	Dislocation of other and unspecified parts of neck
S13.4	Sprain of ligaments of cervical spine
S14.0	Concussion and edema of cervical spinal cord
S14.1	Other and unspecified injuries of cervical spinal cord
S22.0	Fracture of thoracic vertebra
S23.1	Subluxation and dislocation of thoracic vertebra
S24.0	Concussion and edema of thoracic spinal cord
S24.1	Other and unspecified injuries of thoracic spinal cord
S32.0	Fracture of lumbar vertebra
S33.1	Subluxation and dislocation of lumbar vertebra
S34.0	Concussion and edema of lumbar and sacral spinal cord
S34.1	Other and unspecified injury of lumbar and sacral spinal cord
S34.3	Injury of cauda equina
T06.0	Injuries of brain and cranial nerves with injuries of nerves and spinal cord at neck level
T06.1	Injuries of nerves and spinal cord involving other multiple body regions
T09.3	Injury of spinal cord, level unspecified
T91.3	Sequelae of injury of spinal cord

Abbreviations: ICD-10 = International Classification of Diseases 10th Revision; SCI = spinal cord injury.
*Wildcard denoting additional digits

Table S2. Flowsheet and medical order codes for identifying CIC and IUC

**Bladder management method**	**Flowsheet codes**	**Medical order codes**
CIC	R OR OUTPUT URINE STRAIGHT CATH [107000511]<br>R INTERMITTENT/STRAIGHT CATH (ML) [3040102830]	PRO150 BLADDER CATHETERIZATION<br>NUR9023 STRAIGHT CATHETERIZATION URINE”
IUC	R POST CATH VOID (ML) [3040102774]	NUR214 INSERT INDWELLING URINARY CATHETER (IUC)<br>NUR73 INDWELLING URINARY CATHETER CARE<br>NUR701 IUC DISCONTINUE – PROVIDER DIRECTED
Non-specific	R URINE OUTPUT [304550]<br>URINE OUTPUT [61]	

Abbreviations: CIC = clean intermittent catheterization; IUC = indwelling urinary catheter.

Table S3. ICD-10 diagnosis codes for UTI definition

ICD-10 diagnosis code	Code description
A49.9	Bacterial infection, unspecified
B96.20	Unspecified Escherichia coli [E. coli] as the cause of diseases classified elsewhere
B96.4	Proteus (mirabilis) (morganii) as the cause of diseases classified elsewhere
B96.89	Other specified bacterial agents as the cause of diseases classified elsewhere
N30	Cystitis
N30.0*	Acute cystitis
N30.2*	Other chronic cystitis
N30.8*	Other cystitis
N30.9*	Cystitis, unspecified
N34*	Urethritis and urethral syndrome
N39.0	Urinary tract infection, site not specified
R31.9	Hematuria, unspecified
T83.511*	Infection and inflammatory reaction due to indwelling urethral catheter

Abbreviations: ICD-10 = International Classification of Diseases 10th Revision; UTI = urinary tract infection.
*Wildcard denoting additional digits

Table S4. Odds ratios of predictors for guideline-discordant CIC

	**Odds ratio**	**95% CI**	***P* value**
Race			
White	Reference		
Asian	0.41	0.13-1.28	*0.13*
Black	0.99	0.75-1.31	*0.94*
Other	1.46	0.94-2.25	*0.09*
Age group, years			
<30	Reference		
31-50	1.07	0.74-1.53	*0.72*
51-64	1.17	0.79-1.71	*0.44*
65-79	1.36	0.86-2.14	*0.19*
≥80	1.16	0.69-1.95	*0.58*
Sex			
Female	Reference		
Male	**1.34**	**1.03-1.73**	***0.03***
IUC duration prior to CIC, days			
0	Reference		
1-11	0.82	0.66-1.02	*0.08*
≥12	**0.65**	**0.49-0.84**	***0.001***
SCI type			
Thoracolumbar level	Reference		
Cervical level	1.18	0.88-1.57	*0.26*
CIC timing			
Nighttime (7pm-7am)	Reference		
Daytime (7am-7pm)	1.07	0.97-1.18	*0.19*
Insurance type			
Private insurance	Reference		
Managed Care	**2.05**	**1.18-3.54**	***0.01***
Medicaid	1.49	0.91-2.43	*0.12*
Medicare	1.62	1.00-2.62	*0.05*
Intravenous fluid infusion	0.86	0.73-1.01	*0.06*

Abbreviations: CI = confidence interval; CIC = clean intermittent catheterization; IUC = indwelling urinary catheter; SCI = spinal cord injury.
